# The Diet Treatment of Decayed Teeth

**Published:** 1871-08

**Authors:** 


### SELECTED ARTICLES.

---

### ARTICLE IV.

### *The Diet Treatment of Decayed Teeth.*

Dr. J. M. Porter discusses this subject in the *Dental Register*, as follows:

"It would seem from the many articles published in dental journals, advocating the use of phosphate of lime, in all cases where that constituent seems to be deficient in quantity in the teeth, that many dentists regard this as the only successful trèatment in all such cases. And believing that many are laboring without securing the desired results in using the remedy, we will endeavor to give a reason for their want of success.

"The observing dentist, who never makes an operation in the mouth, without examining closely the cause of decay, will many times see that constitutional defects are having as much influence in producing decay, as such local influence, that are sometimes present.

"It is well known that loss and reproduction is constantly going on in the body, that whether formatoin or destructive assimulation predominates, will depend upon general systematic conditions, and *not* upon the amount of any *single element* taken as food. Scientific investigations prove that a given amount of carbon, nitrogen and hydrogen taken as food into the organism will not produce the same amount of physical force in different persons. We may go further and presume that the same amount of food is equally digested in both cases, and absorbed into the circulation and carried into the tissues, and even then we know that the same benefits are not derived in all cases. And further, it is conceded that any kind of nutriment entering the tissue is not proof that it all enters the cells where the greatest amount of oxidation takes place. If, then, a portion of our daily food enters the portals of the tissues without entering the inner chambers of tissues, but passes out again unchanged by a reversal of the forces that carried it in, thereby becoming dead instead of living matter, will giving phosphate of lime have any beneficial influence, supposing this element seems to be deficient in the teeth? Can you relieve such a condition by giving a single element of food, when nature refuses to partake of the food already digested? On the other hand we will suppose that excessive waste is being carried on to such an extent that any part of the body suffers as a result. Will simply giving a single article of food have any influence in checking this excessive waste? If so, upon what principle does it operate?

"From observation and experiment, we have found that proper food (that is, such food as contains the elements which seem to be deficient in quantity), if used judiciously, will remedy a defect of this kind, *if any change of diet can.* If nature refuses to appropriate *food containing such elements as are deficient*, how can we expect better results when we give a single element of food?

"Every dentist who is observing will see a marked difference in the structure of teeth. as he operates in the same mouth for two or three successive years, when the patient has been living on the same diet, and in the same climate, and seemingly in perfect health all the time, and when frequent tests convince the dentist that no local influences are present that could produce these changes.

"We can only account for these changes by presuming that at one time nature refused or failed to appropriate the food digested, for we surely cannot attribute it to any change of diet, when there was no such change.

"In conclusion we claim that it is impossible to modify general systematic conditions by giving as food a single element, which seems to be deficient in some parts of the body. We cannot force nature to appropriate food by such treatment, neither can we check an excessive waste. This thing of treating symptoms is in our opinion very much like reporting personal experiences at dental meetings, neither having the desired influence."

